# Mixed order single variable intuitionistic fuzzy time series forecasting method based on a new artificial neural network and grey wolf optimization algorithm

**DOI:** 10.1038/s41598-026-45059-2

**Published:** 2026-04-02

**Authors:** Turan Cansu, Eren Bas, Erol Egrioglu

**Affiliations:** 1 Department of Statistics, Faculty of Arts and Science, Marmara University, Istanbul, 34722 Turkey; 2 Department of Data Science and Analytics, Faculty of Arts and Science, Giresun University, Giresun, 28200 Turkey

**Keywords:** Intuitionistic fuzzy time series, Artificial neural networks, Dendritic neuron model artificial neural network, Grey wolf optimization algorithm, Forecasting, Engineering, Mathematics and computing

## Abstract

The ease of use of fuzzy time series methods and their success in forecasting performance have led to a rapid increase in research in this field. While classical fuzzy time series methods operate solely on membership values, intuitionistic fuzzy time series methods are based on both membership and non-membership values. In this study, a new mixed-order single-variable intuitionistic fuzzy time series forecasting method is proposed. The proposed approach integrates an artificial neural network, the intuitionistic fuzzy c-means algorithm, and the grey wolf optimization algorithm. The intuitionistic fuzzy time series is constructed using crisp values, membership degrees, and non-membership degrees. Fuzzy relationships are determined through a novel artificial neural network based on the dendritic neuron model and optimized using the grey wolf optimization algorithm. Forecasting models are developed separately based on membership and non-membership values, and the final forecasts are obtained by combining these models using weights determined by the grey wolf optimization algorithm. The performance of the proposed method is evaluated and compared with several existing fuzzy time series methods from the literature using different real-world time series datasets.

## Introduction

In the process of anticipating future events, forecasts can be generated using past and present information. The utilization of historical data assists researchers in making informed decisions under uncertainty. Researchers operating in decision-making environments are required to form more consistent and reliable judgments by explicitly considering uncertainty when forming expectations about the future. Therefore, the combined use of past and present information provides a solid foundation for forecasting. Classical time series methods, fuzzy time series approaches, intuitionistic fuzzy time series methods, fuzzy inference systems, and artificial neural networks are among the most widely used techniques for addressing forecasting problems. Since artificial neural networks can be adapted to model both linear and nonlinear time series, they have become one of the most preferred approaches in the forecasting literature. Despite their strong forecasting capabilities, artificial neural network methods also suffer from certain limitations. Feed-forward neural networks, one of the most commonly used types of artificial neural networks, have been successfully applied to a wide range of time series forecasting problems. The dendritic neuron model artificial neural network (DNM-ANN), proposed by Todo et al.^1^,, is a feed-forward neural network architecture that incorporates both additive and multiplicative aggregation functions. In DNM-ANNs, the flow of input signals is processed sequentially through the synaptic layer, dendritic layer, membrane layer, and soma layer.

The primary function of the synaptic layer, which constitutes the first layer of DNM-ANNs, is to compute the synaptic outputs that are subsequently transmitted to the dendritic layer. The outputs from different synaptic connections are combined in the dendritic layer using a multiplicative operator and then forwarded to the membrane layer. In the membrane layer, signals originating from multiple dendritic branches are aggregated through an additive operator. Finally, the soma layer produces the overall output of the network. As with many artificial neural network models, the training process plays a critical role in determining the performance of DNM-ANNs. In the original study by Todo et al.^1^,, the backpropagation (BP) algorithm was employed to train the DNM-ANN model.

The tendency of the backpropagation (BP) algorithm to become trapped in local minima, together with its relatively slow convergence speed, has led to a decline in its popularity. Consequently, various artificial intelligence–based optimization algorithms have been increasingly employed for network training in recent years. In the literature, numerous studies have proposed and applied DNM-ANNs to forecasting problems, in which different artificial intelligence optimization algorithms have been utilized during the training phase of the network. The optimization algorithms employed for training DNM-ANNs in these studies are summarized in Table 1.

**Table 1:** The various optimisation algorithms used in the training of DNM-ANNs.

Method	Training Algorithm
Zhou et al.^2^,	BP
Yu et al.^3^,	BP
Wang et al.^4^,	Biogeography-based optimization
(Wang et al.^5^, a)	Differential evolution algorithm
(Wang et al.^5^, b)	States Of matter search
Song et al.^6^,	BP
Xu et al., [35]	Whale optimization
Tang et al.^7^,	Artificial immune system
He et al.^8^,	BP
Al-Qaness et al.^9^,	Seagull optimization algorithm
Egrioglu et al.^10^,	Particle swarm optimization
Egrioglu et al.^11^,	Particle swarm optimization
Yilmaz and Yolcu^12^,	Modified particle swarm optimization
Nayak et al.^13^,	Chemical reaction optimization
Cao et al.^14^,	Adam optimizer
Gul et al.^15^,	Sine cosine optimization

The fact that classical time series methods, one of the popular forecasting methods, contain some assumptions such as linearity, model selection, stationarity and number of observations has led researchers to use fuzzy time series methods that do not require such assumptions. Fuzzy time series methods are forecasting methods that utilise the concept of fuzzy logic. Fuzzy logic is based on fuzzy set logic, which is based on the idea that something can be partially true. Although a fuzzy set is based on the idea that the set elements have partial membership, this idea is not entirely suitable for real life. Because it is as important that an element does not belong to the set as it belongs to the set. The hesitation degree of that element belonging to the set is also very important. Based on these ideas, an intuitionistic fuzzy set was proposed by Atanassov^16^as an alternative to a fuzzy set. An intuitionistic fuzzy set contains more information than a fuzzy set considering the above-mentioned features. Therefore, an intuitionistic fuzzy set is a generalisation of a fuzzy set. Thus, intuitionistic fuzzy time series is also a generalisation of fuzzy time series. Various studies are using intuitionistic time series in the literature. Kumar and Gangwar^17^, used the notion of intuitionistic fuzzy time series to handle the non-determinism in time series forecasting. Wang et al.^18^, proposed a novel intuitionistic fuzzy time series forecasting model that the forecast rules were established based on intuitionistic fuzzy approximate reasoning. Lei et al.^19^, proposed a multi-factor high-order intuitionistic fuzzy time series forecasting model. Fan et al.^20^, proposed a long-term intuitionistic fuzzy time series forecasting model based on vector quantisation and curve similarity measure. Fan et al.^21^, proposed an intuitionistic fuzzy time series forecasting model based on order decision and adaptive partition algorithms. Kocak et al.^22^, proposed a new deep intuitionistic fuzzy time series forecasting method based on long short-term memory. Pattanayak et al.^23^, proposed a novel probabilistic intuitionistic fuzzy time series forecasting model using a support vector machine. Chen et al.^24^, proposed a weighted intuitionistic fuzzy time series model based on the quantile discretization approach. Pattanayak et al.^23^, proposed a novel probabilistic intuitionistic fuzzy set with support vector machine modelling. Arslan and Cagcag Yolcu^25^proposed an intuitionistic fuzzy time series forecasting model based on a sigma-pi neural network. Bas et al.^26^, proposed a novel intuitionistic fuzzy time series method based on a bootstrapped combined pi-sigma artificial neural network. Nik Badrul Alam et al.^27^, compared the forecasting performance of the intuitionistic fuzzy time series with different interval lengths. Çakır^28^, proposed a hybrid methodology that combines the intuitionistic fuzzy time series method with the intuitionistic fuzzy c-means method for renewable energy generation forecasting of Turkey. Pant et al.^29^, proposed a computational-based partitioning and strong-cut-based novel intuitionistic fuzzy time series forecasting method. Yücesoy et al.^30^, proposed a new intuitionistic fuzzy time series method based on the bagging of decision trees and principal component analysis. Kocak et al.^31^, proposed a new explainable robust high-order intuitionistic fuzzy time-series method for forecasting. Cagcag Yolcu and Yolcu^32^proposed a cascade-based intuitionistic fuzzy time series prediction model for forecasting problems. Dixit and Jain^33^, proposed an intuitionistic fuzzy time series forecasting method for non-stationary time series. Pabuccu and Barbu^34^, investigated the use of feature selection with annealing, and applied the least absolute shrinkage and selection operator (Lasso) method for forecasting financial time series.

An intuitionistic fuzzy time series is characterised by crisp values, memberships and non-memberships. Unlike fuzzy time series, intuitionistic fuzzy time series methods consider non-membership values in addition to membership values. In this study, a new intuitionistic fuzzy time series forecasting method is proposed. The proposed new method is based on intuitionistic fuzzy clustering, DNM-ANNs and grey wolf optimisation algorithm methods. These membership and non-membership values, which are used as inputs of the proposed method, are determined by the intuitionistic fuzzy clustering method and fuzzy relations are determined by DNM-ANNs based on the grey wolf optimisation algorithm. This new proposed method is a mix of two different DNM-ANN methods. Firstly, a forecast is obtained with the crisp values and membership values of the system, simultaneously another forecast is obtained with the crisp values and non-membership values of the system and these two forecasts are combined with weights optimised by the grey wolf optimisation algorithm. The performance of this new proposed method is evaluated over closing prices of Bitcoin, Crude oil, Euro-USD parity and Gold-adjusted time series.

## Fundamental methods

In this section, a brief presentation of intuitionistic fuzzy sets, intuitionistic fuzzy clustering and grey wolf optimisation algorithm is given respectively.

### Intuitionistic fuzzy sets

While the fuzzy sets proposed by Zadeh^35^ are a general form of the classical set, the intuitionistic fuzzy sets proposed by Atanasov (1986) are a more general form of the fuzzy set. $\:U$ being a universal set, the intuitionistic fuzzy set $\:IF$ is shown as in Eq. (1).

1 $$\:IF=\left\{<x,{\mu\:}_{IF}\left(x\right),{\nu\:}_{IF}\left(x\right)>/x\in\:U\right\}$$

$\:{\mu\:}_{IF}\left(x\right)$ and $\:{\nu\:}_{IF}\left(x\right)$ are memberships and non-memberships functions of the intuitionistic fuzzy set. The following inequality is always satisfied for the membership and non-membership values of the intuitionistic fuzzy set.

2 $$\:0\le\:{\mu\:}_{IF}\left(x\right)+{\nu\:}_{IF}\left(x\right)\le\:1$$

Another important concept in intuitionistic fuzzy sets is hesitation degree. Hesitation degree is directly related to membership and non-membership values and is defined as follows.

3 $$\:{\pi\:}_{IF}\left(x\right)=1-{\mu\:}_{IF}\left(x\right)-{\nu\:}_{IF}\left(x\right)$$

### Intuitionistic fuzzy c-means algorithm

The intuitionistic fuzzy c-means (IFCM) method has an algorithm that can also determine the degrees of membership, non-membership and hesitation for each element of the universal set while clustering the data set. Although there are several different algorithms of IFCM in the literature, the algorithm presented in Chaira^36^ is often preferred.

#### Algorithm 1 Intuitionistic fuzzy c-means clustering algorithm

Step 1. $\:{\mu\:}_{ik}\:,\:i=\mathrm{1,2},\dots\:,c;k=\mathrm{1,2},\dots\:,n$ is the membership value of kth data in ith cluster. Initial values for membership values are randomly generated from a continuous uniform distribution.
Step 2. Using Eqs. (4) and (5), hesitation degrees ($\:{\pi\:}_{ik}$) and intuitionistic memberships ($\:{\mu\:}_{ik}^{\mathrm{*}})$ are calculated.
  4 $$\:{\boldsymbol{\pi\:}}_{\boldsymbol{i}\boldsymbol{k}}\:=1-{\boldsymbol{\mu\:}}_{\boldsymbol{i}\boldsymbol{k}}-{\left(1-{{\boldsymbol{\mu\:}}_{\boldsymbol{i}\boldsymbol{k}}}^{\boldsymbol{\alpha\:}}\right)}^{1/\boldsymbol{\alpha\:}},\:\:\:\boldsymbol{\alpha\:}\:>0$$
  5 $$\:{\boldsymbol{\mu\:}}_{\boldsymbol{i}\boldsymbol{k}}^{\mathbf{*}}={\boldsymbol{\mu\:}}_{\boldsymbol{i}\boldsymbol{k}}+{\boldsymbol{\pi\:}}_{\boldsymbol{i}\boldsymbol{k}}$$
Step 3. $\:{x}_{k}$ is the *k*th data set, $\:{d}_{ik}$ is the Euclidean distance between the *k*th data set and the *i*th cluster centre and $\:m$ is the fuzziness index, and the cluster centres and membership values are recalculated by applying Eqs. (6) and (7).
  6 $$\:{v}_{i}=\frac{\sum\:_{k=1}^{n}{\left({\mu\:}_{ik}^{\mathrm{*}}\right)}^{m}{x}_{k}}{\sum\:_{k=1}^{n}{\left({\mu\:}_{ik}^{\mathrm{*}}\right)}^{m}}\:\:;i=\mathrm{1,2},\dots\:,c$$
  7 $$\:{\mu\:}_{ik}=\frac{1}{{\sum\:}_{j=1}^{c}{\left(\frac{{d}_{ik}}{{d}_{jk}}\right)}^{2/(m-1)}}\:\:\:i=\mathrm{1,2},\dots\:,c;k=\mathrm{1,2},\dots\:,n$$
Step 4. Using Eqs. (4) and (5), the degrees of hesitation and intuitionistic membership values are recalculated.
Step 5. Stopping criteria is checked. The stopping condition is that a distance measure calculated based on the difference between the intuitionistic membership values calculated in Step 2 and the intuitionistic membership values calculated in Step 5 is smaller than a predefined error tolerance. If the condition is satisfied, stop the algorithm, otherwise go to Step 2.

### Grey wolf optimization

The grey wolf optimisation algorithm (GWOA) is a heuristic optimisation algorithm proposed by Mirjalili et al.^37^, that mimics the leadership structure hierarchy and hunting pattern of grey wolves in nature. Grey wolves live in a pack, in which the wolves are placed according to their characteristics. There are four different types of wolves in a grey wolf pack: alpha, beta, delta and gamma wolves. These wolves are connected to each other in a hierarchical structure. At the top of the hierarchy is the alpha wolf, the leader of the pack. The alpha wolf is the leader of the pack and is responsible for the decision-making process in the pack. The beta wolf is the second in the pack hierarchy and its task is to ensure the command of the wolves in the lower hierarchy. The third wolf in the hierarchy is the delta wolf, which follows the orders of the alpha and beta wolves and commands the omega wolves. The other wolves, recognised as omegas, are tasked with ensuring the safety of the pack and taking positions relative to alpha, beta and delta wolves. A grey wolf optimisation algorithm method has a mechanism for changing the position of wolves in the pack according to the movement of the positions of alpha, beta and delta wolves in the pack. GWOA starts with the determination of the hierarchy of a randomly generated wolf pack. The process continues with prey encirclement and hunting steps to update the hierarchy. The algorithm for GWOA is given in Algorithm 2.

#### Algorithm 2 Grey wolf optimization algorithm

Step 1. Determination of algorithm parameters.
  In this first step, the parameters of the algorithm are determined.
  $\:nw:$ the number of wolves in a wolf pack.
  $\:np:$ the number of positions for each wolf.
  $\:maxitr:$ the maximum number of iterations.
  $\:\epsilon\::$ an error tolerance.
Step 2. Generation of a random wolf pack.
  $\:nw$ random wolves are generated with a uniform random distribution U(0,1), where $\:{x}_{ij}$ denotes the $\:jth$ position of the $\:ith$ wolf $\:(i=\mathrm{1,2},\cdots\:,\:nw;j=\mathrm{1,2},\cdots\:,np).$.
Step 3. Determination of the pack hierarchy.
  In this step, the fitness value of each randomly generated wolf is first obtained and a pack hierarchy is created.
  $\:\alpha\::$ the wolf with the best fitness function value.
  $\:\beta\::$ the wolf with the second best fitness value.
  $\:\:\delta\::$the wolf with the third best fitness value.
  $\:\varOmega\:$ the remaining wolves other than α, β and δ wolves.
Step 4. Encircling the prey.
  The wolf pack, which has the ability to detect and encircle prey, surrounds the prey under the leadership of the alpha wolf. Prey enclosure behaviour of grey wolves is given by Eqs. (8–9).
  8 $$\:\overrightarrow{D}=\left|\overrightarrow{C}{\overrightarrow{X}}_{p}\left(t\right)-\overrightarrow{X}\left(t\right)\right|$$
  9 $$\:\overrightarrow{X}\left(t+1\right)={\overrightarrow{X}}_{p}\left(t\right)-\overrightarrow{A}\overrightarrow{D}$$
  Where, $\:t$ is the current iteration, $\:\overrightarrow{A}$ and $\:\overrightarrow{C}$ are the vector of coefficients, $\:{\overrightarrow{X}}_{p}$ is the position vector of the prey, $\:\overrightarrow{X}$ is the position vector of a grey wolf and is the distance to the prey.
  10 $$\:\overrightarrow{A}=2\overrightarrow{a}{r}_{1}-\overrightarrow{a}$$
  11 $$\:\overrightarrow{C}=2{r}_{2}$$
  $\:{r}_{1}$ and $\:{r}_{2}$ are randomly generated values between $\:U\left(\mathrm{0,1}\right)$. Besides, $\:\overrightarrow{a}$ is calculated as in Eq. (12). In Eq. (12), $\:itr$ shows the current iteration.
  12 $$\:\overrightarrow{a}=2-2\left(\frac{itr}{maksitr}\right)$$
Step 5. Hunting.
  In the hunting step, the position update of all grey wolves is performed. This position update is done through the positions of alpha, beta and delta wolves. $\:\overrightarrow{X}\left(t+1\right)$ be the updated position of each wolf, these updated positions are obtained by Eqs. (13–19).
  13 $$\:{\overrightarrow{D}}_{\alpha\:}=\left|{\overrightarrow{C}}_{1}{\overrightarrow{X}}_{\alpha\:}-\overrightarrow{X}\right|$$
  14 $$\:{\overrightarrow{D}}_{\beta\:}=\left|{\overrightarrow{C}}_{2}{\overrightarrow{X}}_{\beta\:}-\overrightarrow{X}\right|$$
  15 $$\:{\overrightarrow{D}}_{\delta\:}=\left|{\overrightarrow{C}}_{3}{\overrightarrow{X}}_{\delta\:}-\overrightarrow{X}\right|$$
  16 $$\:{\overrightarrow{X}}_{1}={\overrightarrow{X}}_{\alpha\:}-{\overrightarrow{A}}_{1}{\overrightarrow{D}}_{\alpha\:}$$
  17 $$\:{\overrightarrow{X}}_{2}={\overrightarrow{X}}_{\beta\:}-{\overrightarrow{A}}_{2}{\overrightarrow{D}}_{\beta\:}$$
  18 $$\:{\overrightarrow{X}}_{3}={\overrightarrow{X}}_{\delta\:}-{\overrightarrow{A}}_{3}{\overrightarrow{D}}_{\delta\:}$$
  19 $$\:\overrightarrow{X}\left(t+1\right)=\frac{{\overrightarrow{X}}_{1}+{\overrightarrow{X}}_{2}+{\overrightarrow{X}}_{3}}{3}$$
  In these Equations, $\:{\overrightarrow{X}}_{\alpha\:}-\overrightarrow{X}$, $\:{\overrightarrow{X}}_{\beta\:}-\overrightarrow{X}$, $\:{\overrightarrow{X}}_{\delta\:}-\overrightarrow{X}$ represents the distance between any wolf and alpha wolf, beta wolf, delta wolf, respectively.
Step 6. Update the positions of alpha, beta and delta wolves.
  At the end of each iteration, the positions of alpha, beta and delta wolves in the pack are updated according to the fitness function.
Step 7. Check the stopping condition of the algorithm.
  The algorithm is stopped if the $\:maxitr$ is reached or if the difference between the best fitness function values for a given number of consecutive iterations is greater than $\:\epsilon\:$.

## Proposed method

In this study, a new intuitionistic fuzzy time series forecasting method is proposed. The proposed method works based on the definitions of intuitionistic fuzzy time series.

### Definitions of intuitionistic fuzzy time series

Egrioglu et al.^38^ introduced a new intuitionistic fuzzy time series definition. In this definition, intuitionistic fuzzy time series is defined as a multivariate time series. Egrioglu et al.’s^38^ definition is given below:

#### Definition 1

(Intuitionistic Fuzzy Time Series, $\:{IF}_{t}\:$) Let $\:{X}_{t}$ is a time series with real observations. $\:{A}_{1},{A}_{2},\dots\:,{A}_{c}$ are intuitionistic fuzzy sets on universal set. Intuitionistic fuzzy time series is a multivariate time series with real observations, membership and non-membership values for each fuzzy sets.

20 $$\:{IF}_{t}=\left\{{X}_{t},\:{\mu\:}_{{A}_{1}}\left({X}_{t}\right),{\mu\:}_{{A}_{2}}\left({X}_{t}\right),\dots\:,{\mu\:}_{{A}_{c}}\left({X}_{t}\right),{\nu\:}_{{A}_{1}}\left({X}_{t}\right),{\nu\:}_{{A}_{2}}\left({X}_{t}\right),\dots\:,{\nu\:}_{{A}_{c}}\left({X}_{t}\right)\:\right\}$$

Where $\:{\mu\:}_{{A}_{j}}\left({X}_{t}\right)$, $\:{\nu\:}_{{A}_{j}}\left({X}_{t}\right)$ are membership and non-membership values of t^th^ observation to jth intuitionistic fuzzy set and these values can be obtained intuitionistic fuzzy c-means or other intuitionistic fuzzy clustering methods.

The high order single variable intuitionistic fuzzy time series forecasting model given in Egrioglu et al.^38^ is modified in this study and a new forecasting model is obtained and given in Definition 2.

#### Definition 2

(Mixed Order Single Variable Intuitionistic Fuzzy Time Series Forecasting Model) Let $\:{IF}_{t}$ be an intuitionistic fuzzy time series. $\:{A}_{1},{A}_{2},\dots\:,{A}_{c}$ are intuitionistic fuzzy sets on universal set. $\:{\mu\:}_{{A}_{j}}\left({X}_{t}\right)$, $\:{\nu\:}_{{A}_{j}}\left({X}_{t}\right)$ are membership and non-membership values of t^th^ observation to jth intuitionistic fuzzy set. The mixed order model can be defined as following form.

21 $$\begin{gathered} X_{t} = G\left( {X_{{t - 1}} , \ldots ,X_{{t - p}} ,~\mu _{{A_{1} }} \left( {X_{{t - 1}} } \right),\mu _{{A_{2} }} \left( {X_{{t - 1}} } \right),} \right. \hfill \\ \left. { \ldots ,\mu _{{A_{c} }} \left( {X_{{t - 1}} } \right),\nu _{{A_{1} }} \left( {X_{{t - 1}} } \right),\nu _{{A_{2} }} \left( {X_{{t - 1}} } \right), \ldots ,\nu _{{A_{c} }} \left( {X_{{t - 1}} } \right)} \right) + \varepsilon _{t} \hfill \\ \end{gathered}$$

Where G is estimated by using a combined neural network based on dendritic neuron model.

### Mixed order single variable intuitionistic fuzzy time series forecasting method

In this study, a new intuitionistic fuzzy time series method is proposed. The difference of the proposed method from other intuitionistic methods in the literature is that it is based on a mixed order model and has a new combined dendritic neuron model for fuzzy relationship detection. In this artificial neural network architecture, membership and non-membership values are processed in two separate parts. In addition, the outputs produced by the processing of membership and non-membership values are weighted with a parameter whose value is obtained during the optimisation process in the last part. The optimisation process is performed by grey wolf optimisation algorithm which is an effective artificial intelligence optimisation algorithm. The new intuitionistic fuzzy time series forecasting method is given in steps in the algorithm below.

#### Algorithm 3 IFTS-DNM-GW method

Step 1. The following initial parameters of the method are determined.
  $\:c:$ The number of intuitionistic fuzzy sets.
  $\:p:\:$The number of lagged variables for time series.
  $\:m:$ The number of dendrites in dendritic neuron model artificial neural networks.
  $\:wn:$ The number of wolfs in the pack.
  $\:maxitr:$ The number of maxiumum iterations.
Step 2. By applying the intuitionistic fuzzy c-means method to the time series, the $\:{(IF}_{t})$ in Eq. (1) is obtained. For example, when the number of observations is n and the number of fuzzy sets is 2, the intuitionistic fuzzy time series is as follows. As seen in Table 2, the intuitionistic fuzzy time series $\:{(IF}_{t})$ is a multivariate time series consisting of 5 time series.

**Table 2:** Example intuitionistic fuzzy time series.

$\:\boldsymbol{t}$	$\:{IF}_{t}$				
	$\:{X}_{t}$	$\:{\mu\:}_{{A}_{1}}\left({X}_{t}\right)$	$\:{\mu\:}_{{A}_{2}}\left({X}_{t}\right)$	$\:{\nu\:}_{{A}_{1}}\left({X}_{t}\right)$	$\:{\nu\:}_{{A}_{2}}\left({X}_{t}\right)$
$\:1$	$\:{x}_{1}$	$\:{\mu\:}_{{A}_{1}}\left({x}_{1}\right)$	$\:{\mu\:}_{{A}_{2}}\left({x}_{1}\right)$	$\:{\nu\:}_{{A}_{1}}\left({x}_{1}\right)$	$\:{\nu\:}_{{A}_{2}}\left({x}_{1}\right)$
$\:2$	$\:{x}_{2}$	$\:{\mu\:}_{{A}_{1}}\left({x}_{2}\right)$	$\:{\mu\:}_{{A}_{2}}\left({x}_{2}\right)$	$\:{\nu\:}_{{A}_{1}}\left({x}_{2}\right)$	$\:{\nu\:}_{{A}_{2}}\left({x}_{2}\right)$
$\vdots$	$\vdots$	$\vdots$	$\vdots$	$\vdots$	$\vdots$
$\:\boldsymbol{n}$	$\:{x}_{n}$	$\:{\mu\:}_{{A}_{1}}\left({x}_{n}\right)$	$\:{\mu\:}_{{A}_{2}}\left({x}_{n}\right)$	$\:{\nu\:}_{{A}_{1}}\left({x}_{n}\right)$	$\:{\nu\:}_{{A}_{2}}\left({x}_{n}\right)$

**Fig. 1:**
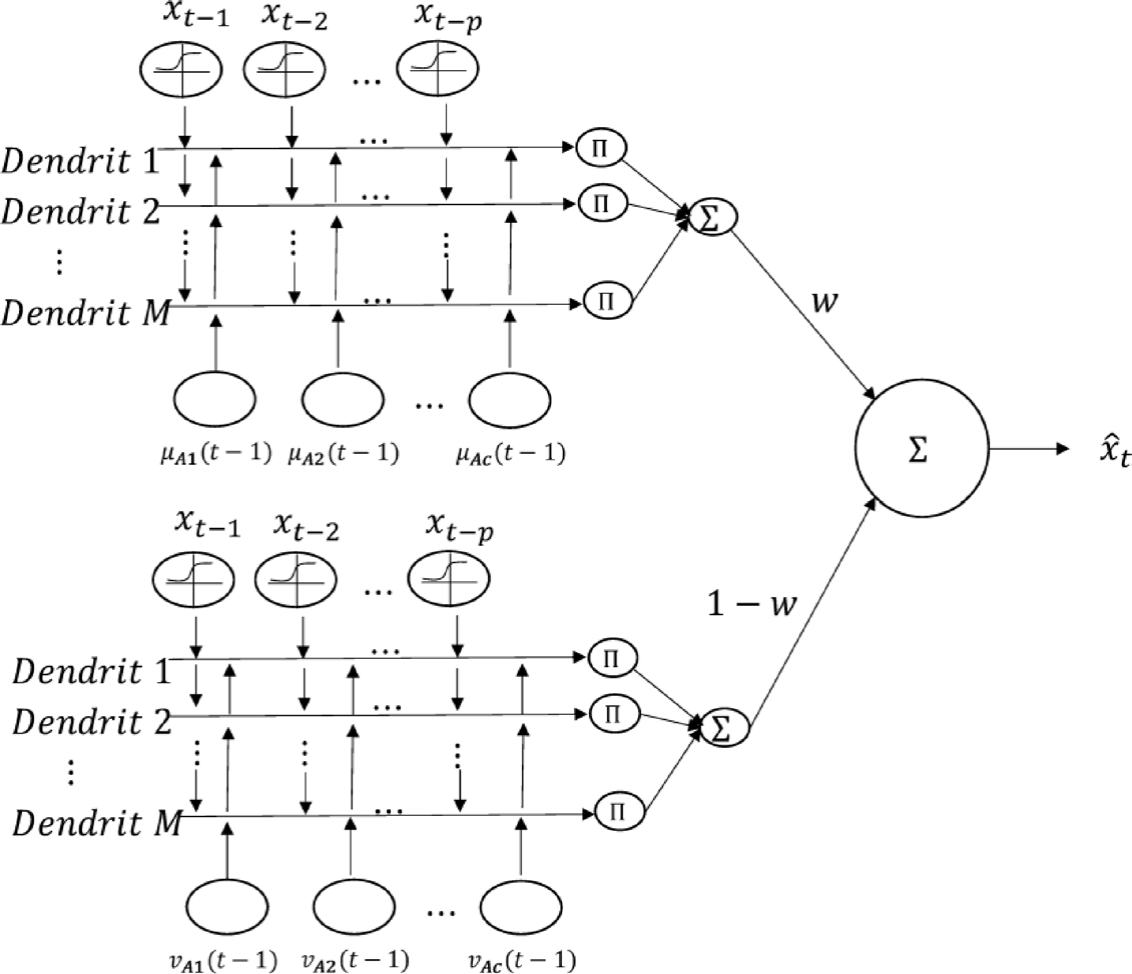
Combined dendritic artificial neural network.

Step 3. The combined dendritic artificial neural network is trained using the grey wolf optimisation algorithm (Algorithm 2). The architecture of the combined dendritic artificial neural network is given in Fig. 1.

The output of the combined dendritic neural network given in Fig. 1 is calculated by the equations given below. The synaptic function output obtained with lagged variables for the first part of the neural network with memberships is given in Eq. (22).

22 $$\:{\mathrm{Y}}_{\mathrm{i}\mathrm{j}}^{1}=\frac{1}{1+\mathrm{e}\mathrm{x}\mathrm{p}(-\mathrm{k}\left({\mathrm{w}}_{\mathrm{i}\mathrm{j}}^{1}{\mathrm{x}}_{\mathrm{t}-\mathrm{i}}+{{\uptheta\:}}_{\mathrm{i}\mathrm{j}}^{1}\right))}\:\:\mathrm{i}=\mathrm{1,2},\dots\:,\mathrm{p};\mathrm{j}=\mathrm{1,2},\dots\:,\mathrm{m}$$

The synaptic function output obtained from the memberships for the first part of the artificial neural network working with memberships is given in Eq. (23).

23 $$\:{Y}_{ij}^{2}=\frac{1}{1+\mathrm{e}\mathrm{x}\mathrm{p}(-k\left({w}_{ij}^{2}{\mu\:}_{{A}_{i}}(t-1)+{\theta\:}_{ij}^{2}\right))}\:\:i=\mathrm{1,2},\dots\:,c;j=\mathrm{1,2},\dots\:,m$$

The output of the synaptic function obtained from the lagged variables for the second part of the neural network, which works with non-membership values, is given in Eq. (24).

24 $$\:{Y}_{ij}^{3}=\frac{1}{1+\mathrm{e}\mathrm{x}\mathrm{p}(-k\left({w}_{ij}^{3}{x}_{t-i}+{\theta\:}_{ij}^{3}\right))}\:\:i=\mathrm{1,2},\dots\:,p;j=\mathrm{1,2},\dots\:,m$$

For the second part of the artificial neural network that works with non-membership values, the synaptic function output obtained from non-membership values is given in Eq. (25).

25 $$\:{Y}_{ij}^{4}=\frac{1}{1+\mathrm{e}\mathrm{x}\mathrm{p}(-k\left({w}_{ij}^{4}{v}_{{A}_{i}}(t-1)+{\theta\:}_{ij}^{4}\right))}\:\:i=\mathrm{1,2},\dots\:,c;j=\mathrm{1,2},\dots\:,m$$

Combined dendritic neuron model artificial neural network has two dendritic layers. The first layer is obtained by Eq. (26) for the first part working with memberships.

26 $$\:{Z}_{j}^{1}=\left(\prod\:_{i=1}^{p}{Y}_{ij}^{1}\right)\left(\prod\:_{i=1}^{c}{Y}_{ij}^{2}\right);j=\mathrm{1,2},\dots\:,m$$

The second layer is obtained by Eq. (27) for the second part working with non-membership values.

27 $$\:{Z}_{j}^{2}=\left(\prod\:_{i=1}^{p}{Y}_{ij}^{3}\right)\left(\prod\:_{i=1}^{c}{Y}_{ij}^{4}\right);j=\mathrm{1,2},\dots\:,m$$

Similarly, there are two membrane layers in the proposed artificial neural network. The output of the first membrane layer is calculated by Eq. (28) and the output of the second membrane layer is calculated by Eq. (29).

28 $$\:{V}^{1}=\sum\:_{j=1}^{m}{Z}_{j}^{1}$$

29 $$\:{V}^{2}=\sum\:_{j=1}^{m}{Z}_{j}^{2}$$

The output of the first part of the combined dendritic neuron model with membership values is calculated by Eq. (30) and the output of the second part with non-membership values is calculated by Eq. (31).

30 $$\:{\widehat{x}}_{t}^{1}=\frac{1}{1+exp\left(-{k}_{soma}^{1}({V}^{1}-\:{\theta\:}_{soma}^{1})\right)}$$

31 $$\:{\widehat{x}}_{t}^{2}=\frac{1}{1+exp\left(-{k}_{soma}^{2}({V}^{2}-\:{\theta\:}_{soma}^{2})\right)}$$

The final output of the combined dendritic neuron model is the weighted combination of the outputs of the working part with membership and non-membership values. This weight is determined automatically for the grey wolf optimisation algorithm in the same way as the other weights. The selection of the Grey Wolf Optimizer (GWOA) for optimizing the weights and thresholds of the Dendritic Neuron Model (DNM) is predicated on its superior balance between exploration and exploitation phases. Unlike meta-heuristic algorithms such as Particle Swarm Optimization (PSO) or Cuckoo Search, GWOA maintains a leadership hierarchy that effectively prevents the model from trapping in local optima—a common challenge in high-dimensional non-linear financial datasets. Furthermore, GWOA is characterized by its computational efficiency and minimal number of control parameters, which reduces the structural risk of over-tuning. In our comparative preliminary tests, GWOA demonstrated faster convergence rates and higher stability in weight optimization for the DNM-ANN architecture compared to traditional gradient-based or other nature-inspired methods.

32 $$\:{\widehat{x}}_{t}=w{\widehat{x}}_{t}^{1}+(1-w){\widehat{x}}_{t}^{2}$$

In this step, the following optimisation problem is solved by grey wolf optimisation.

33 $$\:\underset{{\Xi\:}}{\mathrm{min}}\sum\:_{t=1}^{n}{\left({x}_{t}-\widehat{{x}_{t}}\left({\Xi\:}\right))\right)}^{2}$$

Where $\:{\Xi\:}$ is the vector containing the networks of the combined dendtritic neuron model artificial neural network.

34 $$\begin{gathered} \Xi = \left\{ {\left\{ {\theta _{{ij}}^{1} } \right\},\left\{ {w_{{ij}}^{1} } \right\},\left\{ {\theta _{{ij}}^{2} } \right\},\left\{ {w_{{ij}}^{2} } \right\},\left\{ {\theta _{{ij}}^{3} } \right\},\left\{ {w_{{ij}}^{3} } \right\},\left\{ {\theta _{{ij}}^{4} } \right\},\left\{ {w_{{ij}}^{4} } \right\}} \right., \hfill \\ \left. {k_{{soma}}^{1} ,~\theta _{{soma}}^{1} ,k_{{soma}}^{2} ,~\theta _{{soma}}^{2} ,w} \right\}~,i = 1,2, \ldots ,p,j = 1,2, \ldots ,m \hfill \\ \end{gathered}$$

Step 4. The forecasts of the proposed IFTS method are calculated using the network trained in Step 3.

## Experimental study and results

In practice, the performance of the proposed method is tested using different time series. The time series used in the application are divided into 3 parts as training-validation and test set. With the training set, the parameter estimates of the methods applied for different hyperparameter sets were performed, and with the validation set, the best hyperparameter set was determined. The hyperparameters of the method consist of the lag number (p), the number of fuzzy sets (c), and the number of dendrites (m). In the last stage, the methods for the best hyperparameter set were trained with 30 different random initial weights and the statistics of the test set performance were calculated. For a fairer comparison, the results were compared based on the mean, median, standard deviation, interquartile range, minimum, and maximum statistics in the test results. To reduce the effect of unexpected good or bad results that may arise due to random initializations, all methods were run 30 times with different random initializations to present a fair result. For a more equitable comparison, the results were compared based on the mean, median, standard deviation, interquartile range, minimum, and maximum statistics in the test results.

In the comparison of the performance of the proposed method, the classical fuzzy time series methods Chen^39^ and Chen^40^ fuzzy time series solution methods, the fuzzy time series network (FTS-N) proposed in Bas et al.^41^, the fuzzy time series method based on multiplicative neuron model (FTS-SMN) proposed in Aladag^42^, and the picture fuzzy regression functions method proposed in Bas et al.^43^ were used. All methods were applied under the same experimental setup. All models were tested on the same datasets with identical training/testing splits. Parameters for baseline models were optimized using the same grid search/meta-heuristic effort to ensure a fair comparison. Since Chen^39^ and Chen^40^ methods do not depend on random initial weights, these methods have only one result for the best hyperparameter set and statistical results cannot be obtained for these methods. As in classical time series approaches, there are no assumptions in the application of the methods. However, while none of the applied methods are dynamic, it is assumed that the time series is time invariant.

**Fig. 2:**
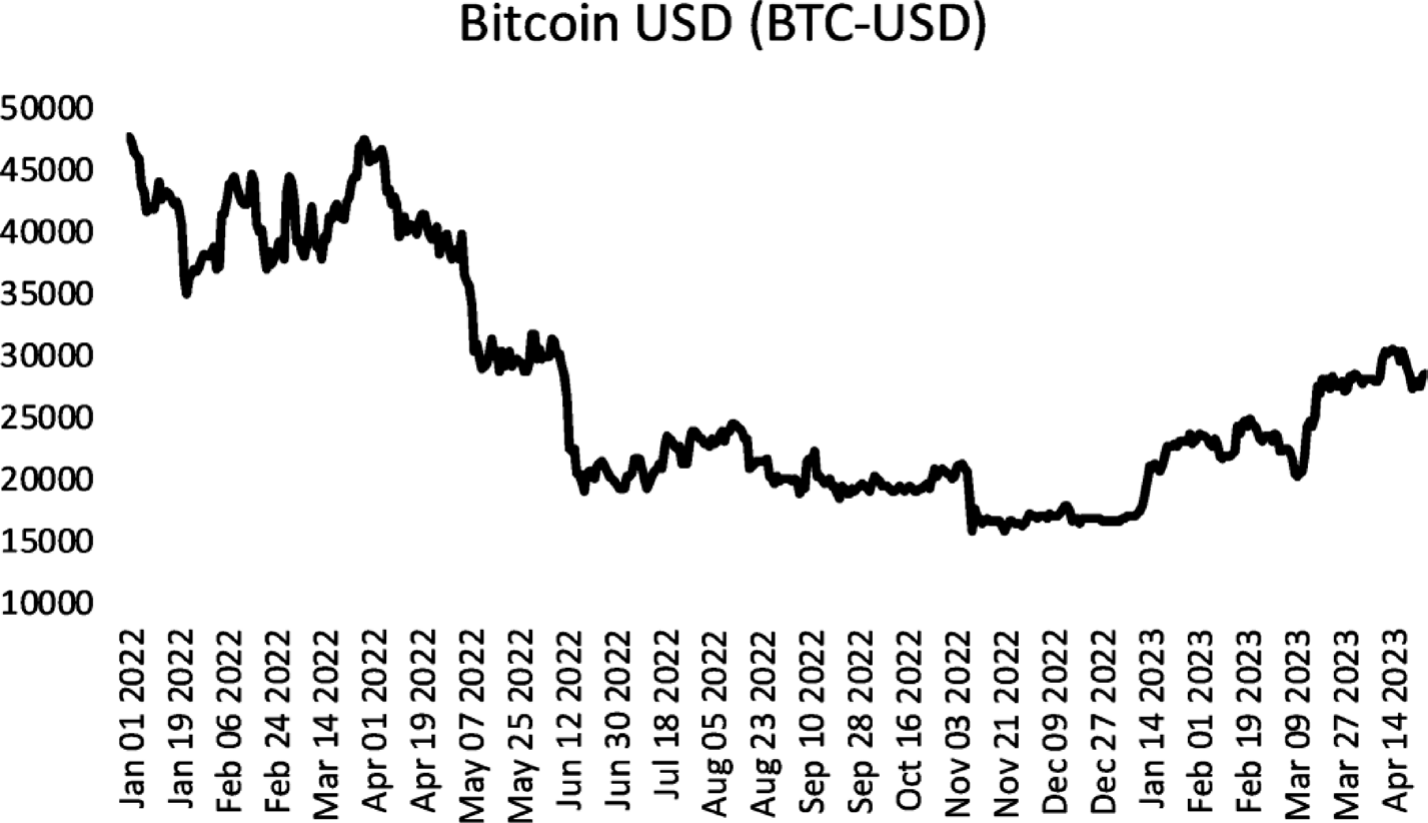
Bitcoin closing prices between 01/01/2022 and 26/04/2023.

The first time series used in the application is the time series of Bitcoin USD (BTC-USD) adjusted closing prices between 01/01/2022 and 26/04/2023. Time series were obtained from “finance.yahoo.com” web site. The time series contains a total of 481 observations, and 14 observations are allocated as validation and test sets, respectively. The graph of the time series is given in Fig. 2. The number of delays in applying all methods varies between 1 and 10, the number of fuzzy clusters varies between 2 and 10, and the number of hidden layer units (number of dendrites in the proposed method) varies between 1 and 5.

The results obtained by analysing the time series are given in Table 3. Table 3 presents the mean, median, interquartile range (IQR), standard deviation, and minimum and maximum statistics of RMSE for the test results obtained for FTS-N, FTS-SMN, PFF and the proposed method for 30 different random initial weights. The results of Chen^39^ and Chen^40^ methods in the best hyperparameter setting are presented.

35 $$\:RMSE=\sqrt{\frac{1}{ntest}\sum\:_{t=1}^{ntest}{\left({x}_{t}-\widehat{{x}_{t}}\right)}^{2}}$$

**Table 3:** Application results for BTC-USD adjusted closing prices.

Methods	Mean	Median	IQR	Std. Dev.	Min	Max	*p*	c	m
Bas et al.^41^	691.5468	692.1735	4.7330	5.4836	682.2478	702.0565	7	2	-
Aladag^42^	675.4390	675.4390	0.0000	0.0000	675.4390	675.4390	2	6	-
Bas et al.^43^	810.0293	713.3952	482.9762	47.3909	665.8274	3359.0710	2	6	-
Chen^39^	988.3330						1	6	-
Chen^40^	781.0575						9	7	-
Proposed Method	639.8515	640.3335	45.1985	63.4928	525.9122	756.2365	3	3	2

Table 3 shows that the mean result of the proposed method is better than all other methods. However, it is understood from the IQR and standard deviation statistics that the proposed method is more affected by random initial values than the other methods. It is also seen that the minimum result of the proposed method shows a very good forecasting performance compared to other methods. The maximum number of the RMSE values for Bas et al.^43^ is shown an extreme value. This value is due to the method’s excessive sensitivity to certain random initial weights.

**Fig. 3:**
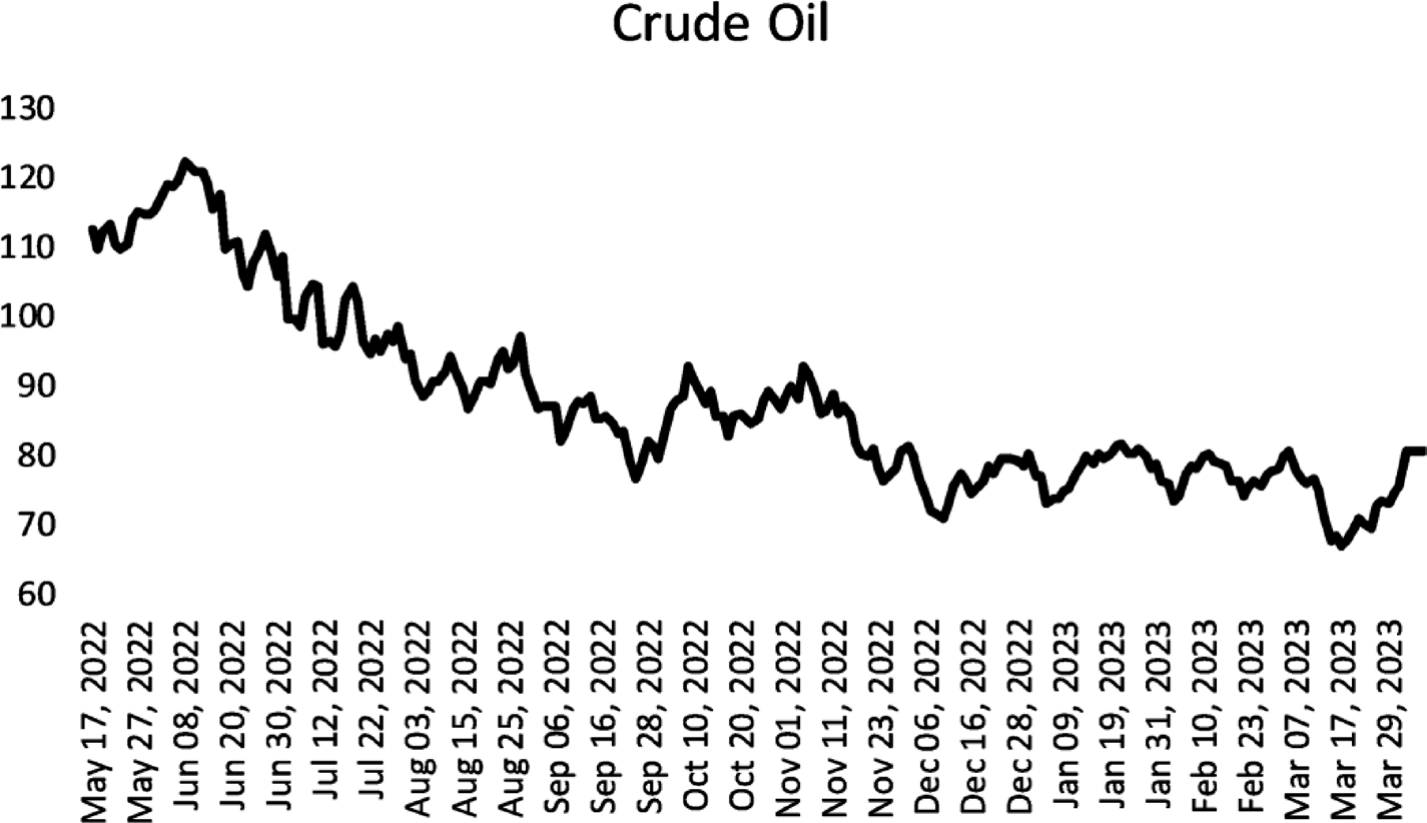
Crude oil adjusted closing prices between 17/05/2022 and 06/04/2023.

The second time series used in the application is the time series of crude oil adjusted closing prices between 17/05/2022 and 06/04/2023. The time series was obtained from finanace.yahoo.com. The graph of the time series is given in Fig. 3.

The results obtained from the analysis of the time series are given in Table 4. As seen in Table 4, the mean statistic result of the proposed method seems to be better than all other methods.

**Table 4:** Application results for crude oil adjusted closing prices.

Methods	Mean	Median	IQR	Std. Dev.	Min	Max	*p*	c	m
Bas et al.^41^	2.0496	2.0425	0.0644	0.0637	1.9498	2.2042	7	7	-
Aladag^42^	2.3435	2.3579	0.0327	0.0000	2.2714	2.3579	7	6	-
Bas et al.^43^	2.1910	2.2282	0.2335	0.3811	1.8530	2.6743	2	5	-
Chen^39^	3.3287						2	7	-
Chen^40^	2.2077						5	6	-
Proposed Method	1.9337	1.8945	0.1122	0.1338	1.7802	2.2186	3	2	2

**Fig. 4:**
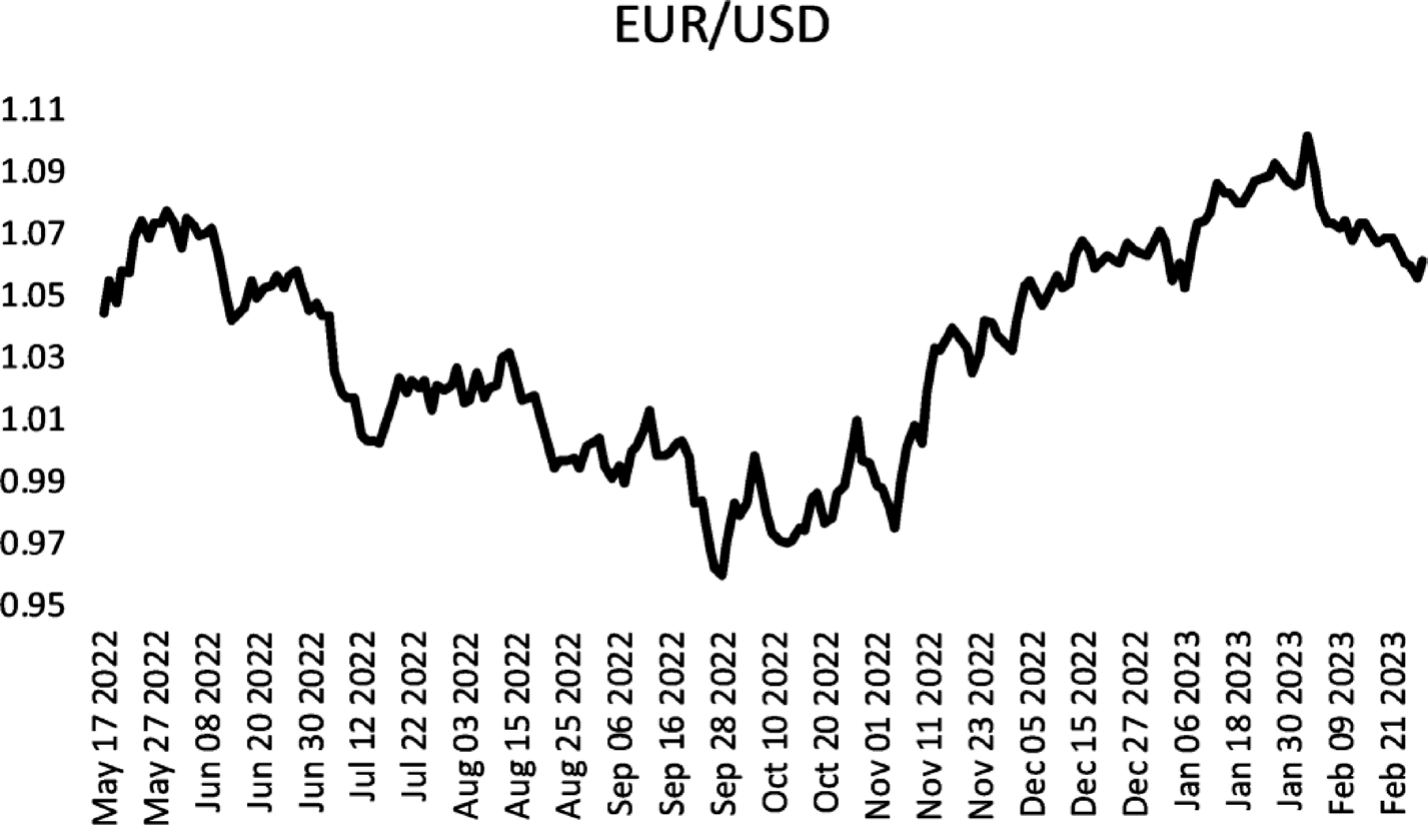
EUR/USD adjusted closing prices between 17/05/2022 and 28/02/2023.

The third time series used in the application is the time series of adjusted closing prices of the Euro-USD parity between 17/05/2022 and 28/02/2023. The time series was obtained from finanace.yahoo.com. Graph of the time series Fig. 4 is given in Fig. 4.

The results obtained from the analysis of the time series are given in Table 5. When Table 5 is analysed, it is seen that the proposed method has the best mean result after Chen^39^, so it can be said that the method is the second-best method for this data set. It is also seen that the minimum result produced by the proposed method is better than Chen^39^.

**Table 5:** Application results for Euro/USD adjusted closing prices.

Methods	Mean	Median	IQR	Std. Dev.	Min	Max	*p*	c	m
Bas et al.^41^	0.0042	0.0041	0.0006	0.0008	0.0032	0.0056	2	5	-
Aladag^42^	0.0042	0.0037	0.0007	0.0013	0.0035	0.0057	5	6	-
Bas et al.^43^	0.0041	0.0040	0.0003	0.0004	0.0036	0.0048	1	9	-
Chen^39^	0.0000						1	7	-
Chen^40^	0.0000						6	4	-
Proposed Method	0.0039	0.0037	0.0005	0.0005	0.0034	0.0052	3	4	3

**Fig. 5:**
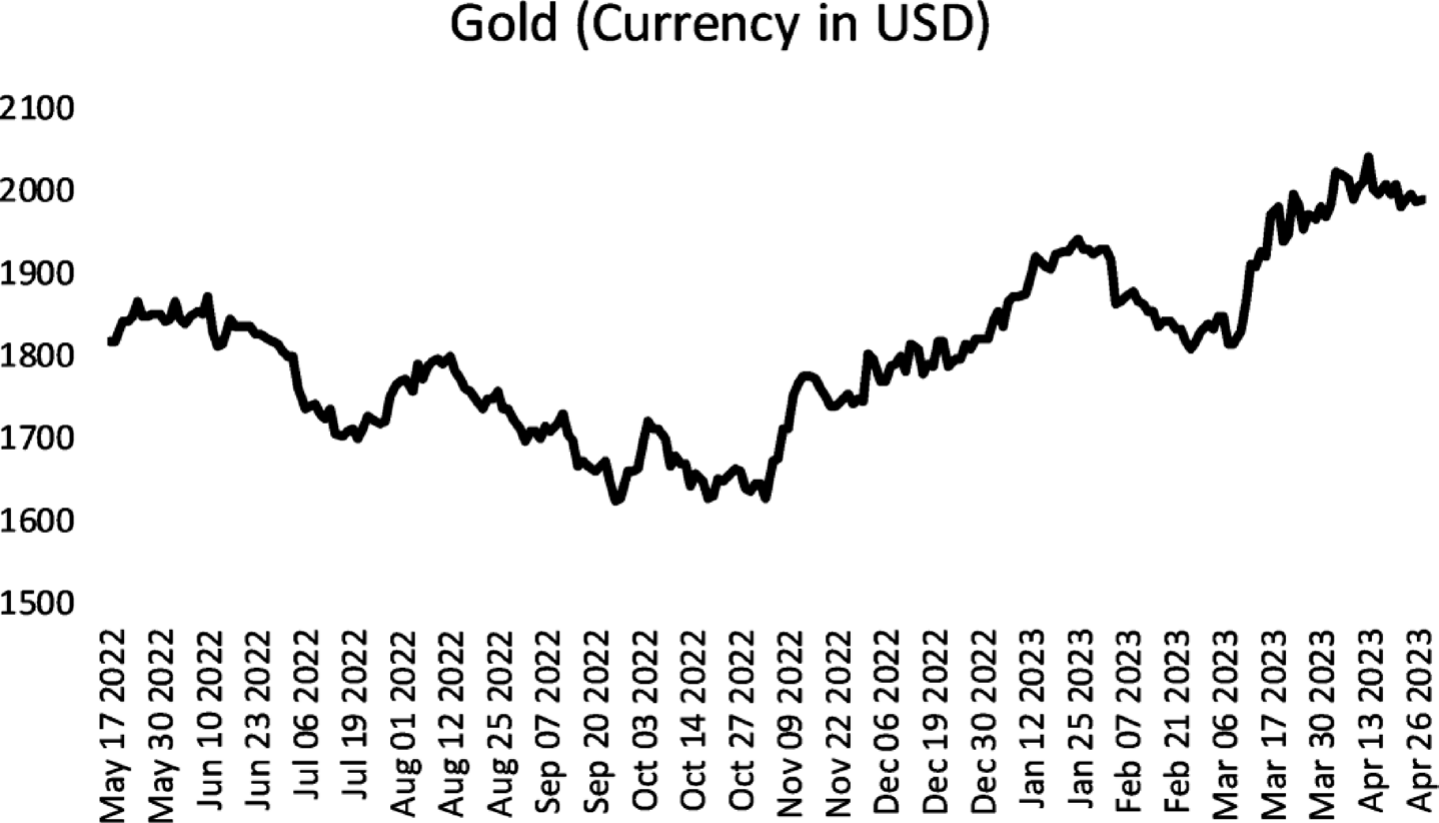
Gold adjusted closing prices between 17/05/2022 and 27/04/2023.

Finally, we analysed the time series of gold (currency in USD) adjusted closing prices between 17/05/2022 and 27/04/2023. The time series is plotted in Fig. 5. The time series was obtained from finanace.yahoo.com.

The results obtained from the application of the methods to the Gold Adjusted Closing Prices time series are given in Table 6. Table 6 shows that the proposed method has the lowest mean RMSE statistic. It is also seen that the proposed method is more successful than all methods in both minimum and maximum statistics.

**Table 6:** Application results for gold-adjusted closing prices.

Methods	Mean	Median	IQR	Std. Dev.	Min	Max	*p*	c	m
Bas et al.^41^	18.7351	18.7063	0.2288	0.3299	18.31782	19.1487	6	5	-
Aladag^42^	19.2404	18.5879	0.9670	1.8123	18.25137	21.6825	4	5	-
Bas et al.^43^	20.1202	19.8325	1.5300	1.9100	17.88667	24.6207	1	9	-
Chen^39^	21.4973						1	7	-
Chen^40^	31.7303						4	3	-
Proposed Method	18.3596	18.5591	0.3314	0.4973	17.6043	18.6233	1	5	2

The computational complexity of the proposed method was evaluated in terms of training time and compared with baseline models. Although the inclusion of the dendritic structure and the GWOA optimization increases the number of trainable parameters compared to a standard MLP (43.45 s), the average training time for the proposed model was recorded as 46.57 s on a [12th Gen Intel(R) Core (TM) i5-12500 H (2.50 GHz), 16DB RAM] processor. This remains well within the acceptable limits for daily or hourly financial forecasting tasks.

## Conclusions and discussion

In this study, a mixed forecasting model for modelling intuitionistic fuzzy time series is defined. While the degree of lagged variables in the forecasting model can be greater than one, only one-step lag of membership and non-membership degrees are taken into account. This is designed to prevent the forecasting model from having an excessive number of inputs. A new combined artificial neural network model is presented for the solution of the proposed forecasting model. In the combined neural network, unlike other heuristic systems, the information from the membership and non-membership values is automatically weighted and combined in the last layer. The optimisation problem is solved by the grey wolf algorithm which is an efficient numerical artificial intelligence optimisation method. The first difficulty encountered can be stated as the need for a new fuzzy time series definition in presenting the proposed method. Creating a combined ANN architecture presented another challenge. Since the optimization of the network is an optimization problem involving many parameters, the grey wolf algorithm was used to solve this problem. The performance of the proposed method is compared with alternative methods from the literature using various financial data. It is observed that the one-step ahead forecasting performance of the method is better than other methods. The proposed method has the advantage of using a combined artificial neural network in the intuitionistic fuzzy time series approach. Thus, unlike other intuitionistic systems, it presents a more compact and effective approach by training a single ANN instead of two separate optimization processes for membership and non-membership values. Another important difference between the proposed method and fuzzy time series approaches in the literature is its mixed order structure. This structure allows the model’s performance to be improved by using only the first lags of the membership values while utilizing a larger number of delays of the time series. The limitation of the proposed method is that it has been proposed for univariate time series analysis.

In future studies, it is possible to integrate the method with bootstrap methods to reduce the variability of the method due to random starts, and thus it can be made into a method that will provide interval forecasts as well as point forecasts.

## Data Availability

The datasets used and/or analysed during the current study are available from the corresponding author on reasonable request.
